# Validation in Spanish and English of the Transgender Inclusive Behavior Scale

**DOI:** 10.1007/s10508-024-02982-7

**Published:** 2024-09-19

**Authors:** Laura Alonso-Martínez, Davinia Heras-Sevilla, María Fernández-Hawrylak, Johannes Hönekopp, Simon Forrest, Shanna Katz Kattari

**Affiliations:** 1 Department of Health Science, Faculty of Health Science, University of Burgos, Paseo de los Comendadores, 09001 Burgos, Spain; 2 Department of Science of Education, Faculty of Education, University of Burgos, Burgos, Spain; 3 Department of Psychology, Faculty of Health and Life Sciences, Northumbria University, Newcastle Upon Tyne, UK; 4 Department of Sociology, Faculty of Arts and Humanities, Durham University, Durham, UK; 5 Department of Women’s and Gender Studies, School of Social Work, University of Michigan, Ann Arbor, MI USA

**Keywords:** Transgender Inclusive Behavior Scale, Diversity, Gender, Transgender, Gender Diverse

## Abstract

The Transgender Inclusive Behavior Scale (TIBS) seeks to measure transgender-inclusive behavior, specifically actions and language use that support transgender people. The TIBS was developed in the United States. This study aimed to develop a Spanish version of the TIBS and confirm the structure of the English version to explore the psychometric properties and evaluate the construct validity in new contexts. We examined predictors of transgender-inclusive behavior by conducting a comparative analysis between participants from Spain and the United Kingdom. The study involved 1,110 university students, with 545 participants hailing from Spain (375 women, 162 men, and 8 non-binary individuals) and 565 participants from the United Kingdom (368 women, 178 men, and 19 non-binary individuals). Exploratory and confirmatory factor analysis were conducted to investigate and validate the factorial structure of the TIBS. The factor analysis results for the 15 items on the scale confirmed a three-dimensional structure in both languages. The scale score reliability was excellent with a Cronbach’s alpha (*α*) = .95 in the British sample and with an *α* = .89 in the Spanish sample. Being a woman, being lesbian, gay, bisexual, trans, queer, intersex, and/or asexual, and being non-religious were the strongest predictors of inclusive behaviors towards transgender people in both countries. The correlations found indicated that people with lower sexual risk behaviors, and lower sexist, homophobic, and transphobic attitudes also presented higher inclusive behaviors towards trans people. These findings support the development of community strategies to increase the social inclusion of transgender people. The TIBS is a useful measure to track their success.

**Supplementary Information:**

The online version contains supplementary material available at 10.1007/s10508-024-02982-7.

## Introduction

### Trans-Inclusive Behaviors

A trans-inclusive approach prioritizes social justice and the inclusion of transgender individuals into the community (Damanpak et al., 2021). It goes beyond combating discrimination to promote prosocial behaviors (Kim et al., 2024; Waite, 2021), defined as actions visibly and invisibly embracing of all people in the community (Ladwig, 2023; Whitfield et al., 2019). Supporting transgender individuals, respecting gender identity, and ensuring equitable treatment are trans-inclusive practices, which alleviate stress, improve well-being, enhance health service access, and boost motivation and productivity (Bouman et al., 2017; Craig et al., 2021; Petronelli & Ferguson, 2022; Puckett et al., 2022; Rosich, 2020; Timmins et al., 2020; Waite, 2021). These behaviors are crucial in diversity and equity initiatives striving for a supportive environment (Ladwig, 2023; Lee et al., 2021).

While organizations emphasize human rights to foster gender diversity acceptance, the lack of research on trans-inclusive behaviors contrasts with numerous scales measuring discrimination and transphobic attitudes (Clark & Hughto, 2020; Day et al., 2019; Goldberg et al., 2019; Hill & Willoughby, 2005; Nagoshi et al., 2008; Páez et al., 2015; Petronelli & Ferguson, 2022; Santos et al., 2017; Walch et al., 2012). Although these scales are valuable in evaluating negative attitudes directed toward transgender individuals, they do not entirely encompass the spectrum of behaviors contributing to establishing a transgender-inclusive environment (Kim et al., 2024; Whitfield et al., 2019). Measuring trans-inclusive behavior is important, as individuals with low transphobia attitudes scores may display varying levels of trans-inclusive behaviors (e.g., speak out for transgender individuals). This distinction helps to evaluate individual acts of inclusive behaviors (Kattari et al., 2018; Ladwig, 2023).

### Development of the Transgender Inclusive Behavior Scale

The Transgender Inclusive Behavior Scale (TIBS), developed by Kattari et al. (2018) in the U.S., is an innovative instrument for assessing individual inclusive behaviors towards transgender individuals. These behaviors may be influenced by policy framework and structural factors. Unlike other instrument, the TIBS was designed for use by individuals of all gender identities, rather than being limited to either cisgender or transgender individuals (Whitfield et al., 2019). Comprising 15 Likert-type items (1 = never to 5 = always), TIBS assesses behaviors such as safeguarding transgender rights, employing gender-inclusive practice language, and understanding gender-inclusive policies in local resources, labor, and housing. Previous research has found high internal consistency (*α* = 0.93) for the scale in its original version, and scores range from 15 to 75 with lower scores indicating less inclusive behaviors (Kattari et al., 2018; Whitfield et al., 2019).

The initial 30 items comprising the Kattari et al. (2018) scale were developed in partnership with transgender individuals and educators. Four transgender activists, including an academic, assessed initial items, excluding three. After cognitive interviews involving three individuals from the general population, the final scale consists of 23 items. The scale was administered virtually and obtained 918 responses (30.4% transgender and 69.6% cisgender). Analyzing the results, eight items were eliminated, resulting in the 15-item scale, and principal component analysis affirmed the existence of one component. Construct validation revealed higher trans-inclusivity scores among trans individuals, lesbian, gay, bisexual, transgender, queer, intersex, and asexual (LGBTQIA+) community members, and women. Additionally, it correlated strongly with self-reported knowledge of the transgender community (Kattari et al., 2018).

An attempt to validate the TIBS Spanish version was developed by Fernández et al. (2020) in a sample of 59 university lecturers. However, they did not adhere to any established translation guidelines and omitted several scale items.

Therefore, the validation of the TIBS remains incomplete because it requires thorough adaptation to different contexts and languages, such as those in Spain and the United Kingdom where this research is being conducted. Achieving this will enable the TIBS to become a standardized measure, reliable and valid in Spain and the United Kingdom. This advancement will also ensure the attainment of the external validity needed to evaluate and compare actions taken individually to support transgender individuals across Spanish- and English-speaking countries.

### Importance of Interculturality for the Validation of the Transgender Inclusive Behavior Scale in the UK and Spain

The validation of a cross-cultural scale involves consideration of multifaceted factors encompassing cultural, historical, political, economic, legislative, educational, and personal realms to ensure instrument relevance and validity in diverse societies (Ferrando et al., 2022; Kim et al., 2024; Lorenzo & Ferrando, 2021; Sakkaphat et al., 2013). This study explores the impact of cultural nuances on the interpretation of TIBS items in Spain and the United Kingdom, emphasizing the influence of country-specific traditions and values on perceptions of gender identities (Cheung & Rensvold, 2002; Whitfield et al., 2019).

Diverse global strategies, both legal and non-legal, shape responses to transgender concerns, necessitating the use of measurement tools to discern the impact of different approaches (Petronelli & Ferguson, 2022). Disparities in governmental policies and legislation regarding transgender rights between the UK and Spain influence societal acceptance and perception of transgender individuals (Alonso-Martínez et al., 2021). Notably, Spain’s Law 2/2021 streamlines gender recognition processes, promoting social equality and combating discrimination (Spanish Ministry of the Interior, 2022). However, challenges persist, as indicated by the SMI’s (2022) reports, which reveal that hate crimes related to sexual orientation and gender constitute 20.12% (1,041 cases) of all hate crimes. Additionally, the latest education law underscores the inclusion of LGBTQIA+ content in all subjects, particularly emphasizing Education in Ethical and Civic Values (Berajano & García, 2016; Cunha et al., 2021).

In the UK, public discourse and proposed amendments to the Gender Recognition Act (2004) aim to foster inclusive behaviors towards transgender individuals, addressing employment opportunities and mitigating transphobic sentiments (Mukoro et al., 2021). However, the recent overturning of Scotland’s Gender Recognition Reform Bill by the UK Government, the Independent Review of Gender Identity Services for Children and Young People (The Cass Review) commissioned in 2020 by National Health Service (NHS) England and a surge in hate crimes against trans individuals underscores the urgency for change, documented by the Government of United Kingdom (2021) as escalating from 2,451 complaints in 2020 to 2,799 in 2021.

Cross-cultural disparities, rooted in varying cultural, social, and political factors, impact the perception of gender, necessitating adaptability of scales for cultural relevance and accuracy in diverse settings (Dong & Dumas, 2020; Putnick & Bornstein, 2016). This comprehensive perspective recognizes the complexity and dynamism of gender, highlighting the importance of adapting scales for cultural relevance and accuracy in diverse settings (Kattari et al., 2018; Whitfield et al., 2019).

### The Present Study

This study addresses limitations in prior research by Kattari et al. (2018). To fill this gap, our study undertakes these analyses to evaluate the TIBS’ construct validity in measuring inclusive behaviors in Spanish and English. Guided by Norton and Herek’s (2013) Transferability Hypothesis, we explore whether prejudice towards diverse sexual orientations correlates with gender discrimination.

Convergent validity is assessed by comparing the TIBS with established scales measuring sexism (Double Standard Scale-DSS; Caron et al., 1993), homophobia (Heterosexual Attitudes Toward Homosexuality Scale-HATH; Larsen et al., 1980), and transphobia (Scale of Negative Attitudes towards Trans People-EANT; Páez et al., 2015) a, all showing good reliability and validity in English and Spanish. Kaufman et al. (2019) also found evidence that trans-inclusive behavior was related to greater communication of sexual behaviors. Consequently, we compared the TIBS to the Health Protective Sexual Communication Scale (HPSC; Catania, 1998).

Our study aims to establish convergent and discriminant validity for TIBS scores, adapt and validate a Spanish version, and confirm the factor structure in the UK. In line with previous literature (Kanamori et al., 2017; Kattari et al., 2018), the study hypothesized that being a woman, being LGBTQIA+, not being religious, and being younger will be related to a higher presence of trans-inclusive behaviors.

## Method

### Participants

A total of 1,110 student from Spanish and UK universities (*M*_age_ = 23.12 years; *SD* = 5.93) participated in this study. Gender was coded with the following options: woman, man, non-binary gender, and other gender (with an open-ended choice). Due to this classification, separate analyses were hindered by insufficient statistical power. A description of the sample by gender, country, and age is presented in Table 1.

**Table 1:** Sample description

	Spain			United Kingdom			Total		
		Age			Age			Age	
		M (SD)	Min–Max		M(SD)	Min–Max		M(SD)	Min–Max
Gender									
Woman^a^	375	24.2 (6.6)	18–53	368	21.8 (4.4)	8–52	743	23.0 (5.8)	18–53
Man^b^	162	25.3 (7.1)	18–65	178	21.5 (4.2)	8–47	340	23.3 (6.0)	18–65
Another gender^c^	8	28.5 (14.1)	20–55	19	21.5 (4.2)	8–32	27	22.1 (4.6)	18–55
Total (100%)	545	24.6 (7.0)	18–65	565	21.7 (4.3)	18–52	1110	23.1 (5.9)	18–65

^a^Includes cisgender and transgender woman

^b^Includes cisgender and transgender man

^c^Non-binary, non-conforming, agender, gender queer or without a response

Other sociodemographic variables of the study (sexual orientation, marital status, religiousness, and university courses) are described in the results section.

### Procedure

This was a cross-sectional study based on a survey via self-completion questionnaire composed of standardized scales. Before the COVID pandemic, university participants were recruited through presentations in classes. During the pandemic, recruitment relied increasingly on university email account, particularly in the U.K. Data were been collected either face to face and online from November 16, 2019 to July 28, 2021. Respondents had to be studying full-time in a Spanish or British university, and be over 18 years old.

In response to constraints identified by Kattari et al. (2018), we conducted a new adaptation. The translators, proficient in both English and Spanish, underwent specialized training in the field of sexuality. The European Social Survey Translation Process was followed, ensuring construct validity. Initial translation, retranslation by experts, and pilot testing with university students and a transgender association were conducted. Feedback on item formulation in Spanish and British English provided valuable contextualization. Both versions of the scale were adapted to each context, with the English TIBS scale is in Appendix 1, and the Spanish TIBS scale is in supplementary materials.

The questionnaire was pilot-tested with 16 experts from academia, politics, schools, and health centers in the two countries. Feedback from LGBTQIA+ associations and 30 students, along with ethical committee input, informed the final survey design.

### Measures

The survey was identical for respondents in Spain and the U.K., in their own languages. The predictor variables were based on sociodemographic details: gender, age, sexual orientation, religiosity, marital status, country, and nationality (local vs. foreign student). The outcome variables were the result of the TIBS and the four following scales:

The HPSC (Catania, 1998) evaluated individuals’ perceptions of verbal interactions with a new sexual partner on safe sex and sexual histories, employing an 8-item questionnaire rated on a 4-point Likert scale (*α* = 0.84). The current study presented acceptable reliability in the British (*M* = 21.45, *SD* = 5.06, *α* = 0.78) and Spanish (*M* = 21.29, *SD* = 4.80, *α* = 0.72) samples.

The DSS (Alonso-Martínez et al., 2024; Caron et al., 1993; Sierra et al., 2007) assesses adherence to the traditional sexual double standard, employing a 10-item questionnaire rated on a 5-point Likert scale (*α* = 0.72). The current study presented high reliability in the British sample (*M* = 43.03, *SD* = 6.52, *α* = 0.86), and questionable in the Spanish sample (*M* = 42.83, *SD* = 4.75, *α* = 0.63).

The HATH (Barrientos & Cárdenas, 2010; Larsen et al., 1980) assesses discrimination against gays and lesbians, employing a 20-item questionnaire rated on a 5-point Likert scale (*α* = 0.92 and Spanish *α* = 0.90). The current study presented adequate reliability in the British (*M* = 27.08, *SD* = 11.58, *α* = 0.95) and Spanish (*M* = 30.03, *SD* = 6.69, *α* = 0.77) samples.

The EANT (Alonso-Martínez et al., 2021; Páez et al., 2015) is used to evaluate negative predispositions towards transgender people, employing a 9-item questionnaire rated on a 5-point Likert scale (*α* = 0.81 and Spanish *α* = 0.90). The current study presented adequate reliability in the British (*M* = 17.24, *SD* = 5.65, α = 0.81), and Spanish sample (*M* = 14.5, *SD* = 5.95, *α* = 0.79) samples.

### Data Analysis

Data were analyzed with IBM SPSS AMOS v.26 (CFA) and IBM SPSS v.27.

To address the objectives, we performed EFA and CFA on British and Spanish samples to assess TIBS’s structural validity. EFA revealed item factor structure, while CFA confirmed it. Subsamples comprised 265 British and 245 Spanish for EFA, and 300 British and 300 Spanish for CFA, ensuring demographic similarity. The sample size, 25 times greater than items, attested to analytical adequacy (Líbano et al., 2019).

The factor analysis employed an oblique solution with promax rotation because the factors correlated with each other and Maximum Likelihood estimation was chosen for its statistical robustness with large item sets (Boateng et al., 2018; Ferrando et al., 2022; Lloret et al., 2017). According to Sáiz et al. (2019), factor saturation below 0.30 is omitted and when items exhibit high loadings on multiple factors, factor with stronger loadings and greater theoretical justification should be prioritized.

In CFA, we assessed model comparisons using the chi-square (*χ*^2^) fit index, which reflects the disparity between models and data covariance. Model fit was also evaluated through the Adjusted Goodness-of-Fit Index (AGFI) which should present values greater than 0.80 and Goodness of Fit Index (GFI), Comparative Fit Index (CFI), Incremental Fit Index (IFI), and Tucker–Lewis index (TLI) all of which should exceed 0.90. Additionally, the Root Mean Square Error of Approximation (RMSEA) should be less than 0.1 and the Minimum Discrepancy per Degree of Freedom (CMIN/DF) should be less than 5 for an acceptable fit (Fabrigar et al., 1999; Hermida et al., 2015; Schumacker & Lomax, 2016; Xia & Yang, 2019).

In cross-country sample comparisons, invariance is usually made to ensure that the measurement tools used in the analysis are consistent across the two samples (Dong & Dumas, 2020). Four levels—configurational, metric, scalar, and strict—impose progressively stringent restrictions on factor loadings and intercepts (Cheung & Rensvold, 2002; Dong & Dumas, 2020). Configurational invariance ensures TIBS measures identical underlying factors in both UK and Spanish samples. Metric invariance assesses consistent relationships between factors and TIBS items across groups. Scalar invariance allows meaningful comparison of mean TIBS scores. Strict invariance signifies full equivalence. According to the criteria of Putnick and Bornstein (2016), invariance is considered adequate if the differences in *ΔCFI* are ≤ 0.01, *ΔRMSEA* are ≤ 0.015, and *ΔSRMR* are ≤ 0.015.

Statistical analyses included Pearson correlations (*r* = 0.10/0.30/0.50) and Student’s *t*-tests adhering to Cohen’s (1988) effect size criteria (*ds* = 0.20/0.50/0.80) were used to address the construct validity aim.

## Results

### Validation of the Transgender Inclusive Behavior Scale: Reliability

Table 2 presents the reliability metrics of British and Spanish TIBS, including means, standard deviation, variances, 15-item correlations, and α values after item deletion. All item-total correlations exceeded 0.30, warranting the retention of all items for cross-cultural consistency. British and Spanish samples presented excellent (*α* = 0.95) and high (*α* = 0.89) scale score reliability, respectively.

**Table 2:** British and Spanish Transgender Inclusive Behavior Scale scale score reliability

Item	Item Mean		Item Standard Deviation		Scale mean (Item deleted)		Var.^a^		CI-T^b^		α^c^	
	UK	Spain	UK	Spain	UK	Spain	UK	Spain	UK	Spain	UK	Spain
1/TIBS1	1.9	1.9	1.0	1.2	35.3	34.8	192.1	135.6	.74	.55	.94	.88
2/TIBS2	2.9	2.8	1.4	1.4	34.4	33.9	187.4	134.1	.66	.48	.94	.88
3/TIBS3	2.2	1.8	1.3	1.2	35.1	34.9	188.7	139.1	.71	.42	.94	.89
4/TIBS4	2.5	2.3	1.5	1.6	34.8	34.5	183.7	130.1	.69	.55	.94	.88
5/TIBS5	2.9	2.7	1.5	1.4	34.3	34.0	186.5	132.6	.69	.57	.94	.88
6/TIBS6	1.6	2.1	1.0	1.5	35.7	34.7	196.5	136.9	.62	.38	.94	.89
7/TIBS7	1.7	1.8	1.0	1.2	35.6	35.0	193.7	136.1	.68	.53	.94	.88
8/TIBS8	2.9	2.8	1.4	1.3	34.4	34.0	186.4	131.4	.72	.65	.94	.88
9/TIBS9	2.4	2.8	1.4	1.7	34.8	33.9	185.2	137.7	.71	.31	.94	.89
10/TIBS10	2.4	2.5	1.3	1.2	34.8	34.3	183.8	131.1	.82	.69	.94	.87
11/TIBS11	3.4	3.0	1.4	1.3	33.9	33.8	182.8	130.1	.78	.68	.94	.87
12TIBS12	3.4	3.4	1.4	1.4	33.9	33.3	184.4	130.1	.78	.64	.94	.88
13/TIBS13	2.3	2.4	1.2	1.2	35.0	34.7	190.6	133.0	.69	.62	.94	.88
14/TIBS14	2.1	2.3	1.2	1.2	35.2	34.5	190.3	131.1	.71	.70	.94	.87
15/TIBS15	2.1	2.2	1.2	1.2	35.2	34.5	190.0	131.6	.72	.68	.94	.88

^*a*^Scale variance (item suppressed)

^*b*^Item-total correlation

^*c*^Cronbach’s alpha (item deleted)

### Exploratory Factor Analysis

In the British and Spanish exploratory subsamples, EFA aimed to identify structures beyond the original 1-component model (Kattari et al., 2018). The Kaiser, Meyer & Olkin index values exceeded 0.80 (0.91 in British, 0.88 in Spanish) and Bartlett Tests were significant (British: *χ*^*2*^ = 1947.67, *df* = 105, *p* < 0.001 and Spanish: *χ*^*2*^ = 1955.25, *df* = 105,* p* < 0.001). These results confirmed the data suitability for factor analysis (Líbano et al., 2019). The British EFA revealed a 2-factor solution (F1: 33% and F2: 28% variance), while the Spanish EFA showed a 3-factor solution (F1: 28%, F2: 21% and F4: 14% variance). The resulting factor matrix is detailed in Table 3.

**Table 3:** Transgender Inclusive Behavior Scale factors structure in the British and Spanish exploratory subsamples

British sample					Spanish sample					
Item	Factors			α	Item	Factors				α
	1	2	Subscale-F			1	2	3	Subscale-F	
TIBS2	.48		Transgender inclusive practices advocacy	.90	TIBS4	.71			Transgender inclusive practices advocacy	.83
TIBS4	.62				TIBS5	.72				
TIBS5	.70				TIBS8	.80				
TIBS8	.80				TIBS9	.35				
TIBS9	.58				TIBS10	.72				
TIBS10	.68				TIBS11	.78				
TIBS11	.83				TIBS12	.76				
TIBS12	.84				TIBS1		.80		Gender inclusive language practices	.80
TIBS13	.51				TIBS2		.58			
TIBS1		.77	Transgender inclusive language practice and policy awareness	.88	TIBS3		.66			
TIBS3		.58			TIBS6		.77			
TIBS6		.82			TIBS7		.80			
TIBS7		.78			TIBS13			.66	Transgender inclusive policy awareness	.91
TIBS14		.63			TIBS14			.74		
TIBS15		.68			TIBS15			.76		

### Confirmatory Factor Analyses

Table 4 displays the CFA indices for three models in the British and Spanish confirmatory subsample. The 1-factor and 2-factor models exhibited inferior fit compared to the 3-factor model.

**Table 4:** Confirmatory factor analysis fit indices in the British and Spanish Transgender Inclusive Behavior Scale

Country	Model	χ^2^	df	CMIN/DF	GFI	AGFI	RMSEA	IFI	TLI	CFI	Δ*χ*2	Δdf
UK	1-Factor	838.62	90	9.32	.73	.64	.17	.78	.74	.78		
	2-Factor	564.65	89	6.34	.78	.75	.13	.86	.84	.86	1F–2F = 273.97**	1
	3-Factor	339.98	87	3.9	.86	.81	.10	.93	.91	.93	2F–3F = 224.67**	2
Spain	1-Factor	801.41	90	8.91	.69	.59	.16	.69	.64	.69		
	2-Factor	665.91	89	7.18	.73	.64	.15	.75	.70	.75	1F–2F = 135.5**	1
	3-Factor	286.77	87	3.3	.88	.84	.09	.91	.89	.91	2F–3F = 379.14**	2

^**^ *p* ≤ .01

**Fig. 1:**
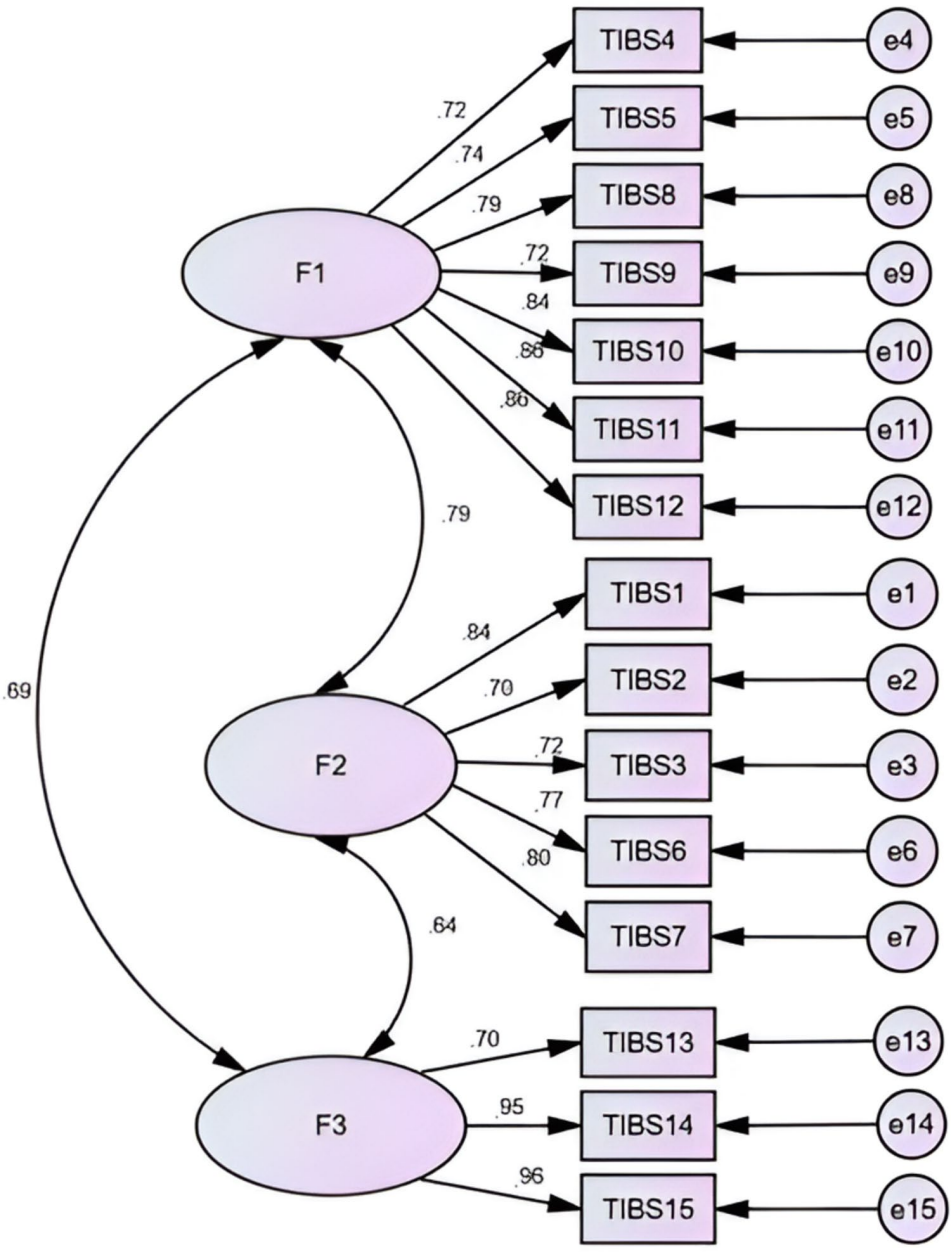
English 3- factor Transgender Inclusive Behavior Scale mode

The British 3-factor model achieved acceptable fit across all seven indicators, (*CMIN/df* = 3.90; *df* = 87, *χ*^2^ = 339.98, *GFI* = 0.86, *AGFI* = 0.81, RMSEA = 0.09, *IFI* = 0.93, *TLI* = 0.91 and *CFI* = 0.93). All effects of factors on items were strong (≥ 0.70) and showed strong positive inter-factor correlations (≥ 0.69, see Fig. 1).

**Fig. 2:**
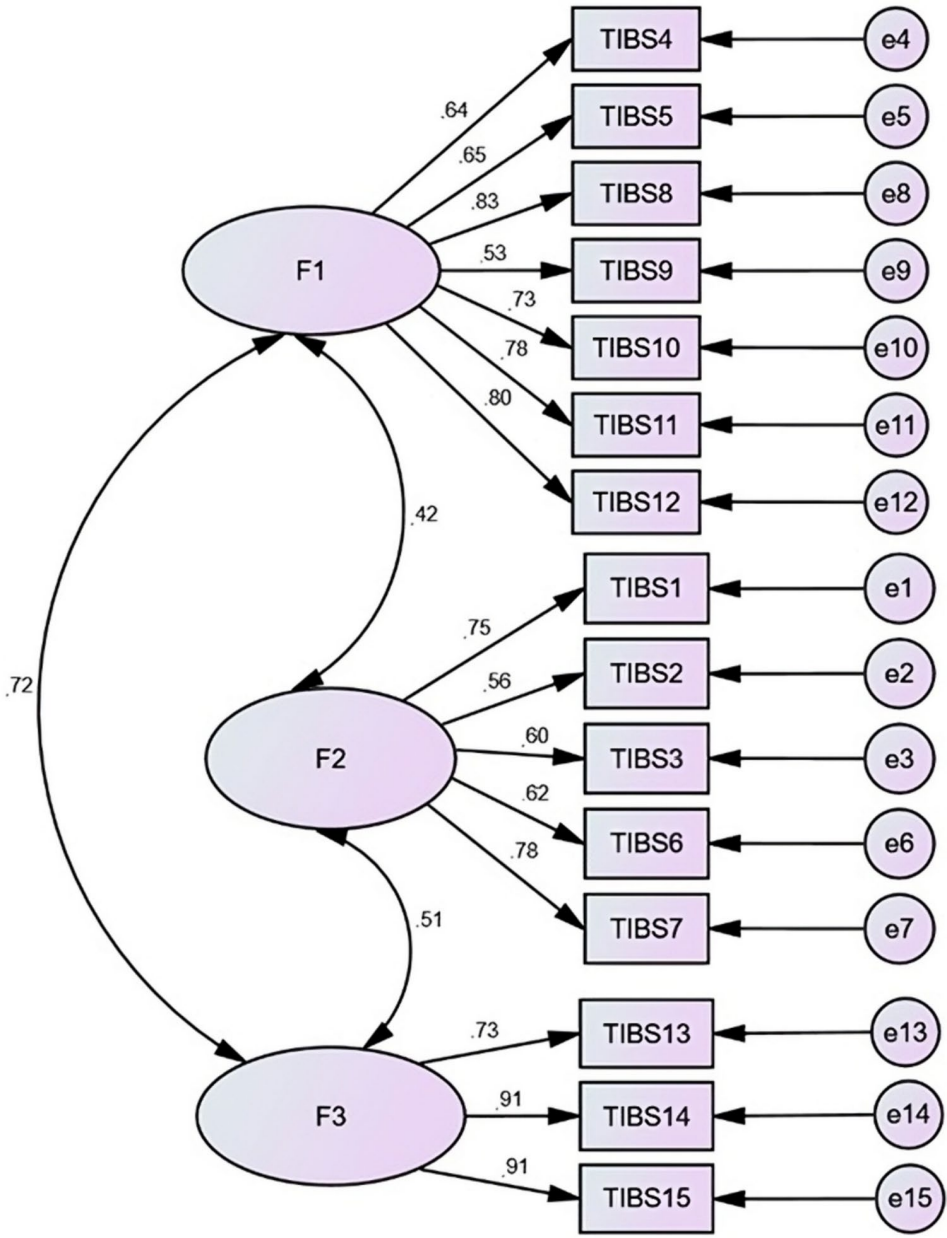
Spanish 3-factor Transgender Inclusive Behavior Scale model

The Spanish 3-factor model achieved acceptable fit across all seven indicators, (*CMIN/df* = 3.21; *df* = 87, *χ*^2^ = 286.77, *GFI* = 0.88, *AGFI* = 0.84, *RMSEA* = 0.08, *IFI* = 0.91, *TLI* = 0.90 and *CFI* = 0.91). All effects of factors on items were strong (≥ 0.56) and showed strong positive inter-factor correlations (≥ 0.42, see Fig. 2).

### Transgender Inclusive Behavior Scale Invariance Between the British and Spanish Confirmatory Subsamples

In this instance, measurement invariance refers to the consistency of TIBS measurements across the two countries. Initially, the configuration invariance model was tested, revealing a trifactorial structure across groups with satisfactory fit indices (*CFI* = 0.92, *RMSEA* = 0.066, *SRMR* = 0.069). Subsequently, the metric invariance model was examined, indicating adequate fit (*CFI* = 0.917, *RMSEA* = 0.065, *SRMR* = 0.076) with minimal differences from the configuration model. Scalar invariance, incorporating equal intercepts, showed a good fit (*CFI* = 0.864, *RMSEA* = 0.080, *SRMR* = 0.081), although unexpected changes in CFI occurred when compared to metric invariance because the *ΔCFI* was > 0.01. The strict invariance model, incorporating restricted error variances, did not fit well (*CFI* = 0.819, *RMSEA* = 0.088, *SRMR* = 0.134), contradicting expectations since *ΔCFI* was > 0.01, the *ΔRMSEA* was > 0.015 and *ΔSRMR* was > 0.015. Comparisons between models were guided by established criteria (Cheung & Rensvold, 2002; Putnick & Bornstein, 2016). When strict invariance is not achieved, it indicates that TIBS is not fully equivalent between the U.K. and Spanish samples. However, according to Dong and Dumas (2020), achieving strict measurement invariance across diverse cultural groups is considered unlikely to be viable due to differences in constructs across languages and populations.

### Descriptive Results of the Transgender Inclusive Behavior Scale and Its Subscales

Table 5 shows the reliability and descriptive statistics of the TIBS and its subscales in the British and Spanish samples.

**Table 5:** Descriptive results of the Transgender Inclusive Behavior Scale and their subscales by samples

	Spanish sample			British sample			Combined sample		
	M (SD)	Min–Max	α	M (SD)	Min–Max	α	M (SD)	Min–Max	α
Subscale 1	16.7 (6.3)	6–30	.87	17.5 (6.8)	6–30	.90	17.1 (6.6)	6–30	.89
Subscale 2	10.4 (4.7)	5–25	.79	10.4 (4.5)	5–25	.85	10.4 (4.6)	5–25	.81
Subscale 3	9.7 (3.9)	4–20	.72	8.9 (4.0)	4–20	.85	9.3 (4.0)	4–20	.79
TIBS scale	36.8 (12.3)	15–75	.89	36.7 (13.9)	15–75	.94	36.7 (13.1)	15–75	.92

### Construct Validity: Comparison of the Transgender Inclusive Behavior Scale with Criterion Variables

To evaluate external validity, we conducted Student’s *t-*tests and correlations between TIBS and its subscales with our study’s criterion variables. The results of the TIBS coincide with those of the subscales. The results in the combined sample were similar to the British and Spanish samples.

The TIBS score (*t*(1108) = -0.061, *p* = 0.95, *d* = 0.004, 95% CI [-0.11, 0.12]) and subscale 2 score (*t*(1108) = 0.02, *p* = 0.80, *d* = 0.02, 95% CI [-0.10, 0.13]) exhibited no significant differences between the British and Spanish samples. However, subscale 1 (*t*(1108) = -2.12, *p* = 0.034, *d* = 0.13, 95% CI [0.01, 0.25]) and subscale 3 (*t*(1108) = 3.39, *p* = 0.001, *d* = 0.20, 95% CI [0.09, 0.32]) showed statistically significant but the effect sizes were small. The British sample exhibited more transgender practice advocacy (subscale 1), whereas the Spanish sample demonstrated more inclusive gender policies (subscale 3). Despite the absence of grammatical norms regulating inclusive language practice in Spanish, no distinctions were observed in subscale 2, which measures the use of gender inclusive language. In addition, Table 6 shows the differences in responses to the TIBS and subscales by gender, sexual orientation, nationality, marital status, and religious affiliation.

**Table 6:** *t*-test for the Transgender Inclusive Behavior Scale and its sub-scales by criterion variables in the British and Spanish samples

Country	Scale	Variables		*t*	df	Sig.	Cohen’s *d*	
							*d*	95% CI
UK		Gender, M (SD)						
		Woman, *n* = 368	Man, *n* = 178					
	Subscale 1	18.5 (6.5)	14.3 (6.3)	6.74	544	.001**	0.61	0.43, 0.79
	Subscale 2	10.8 (4.2)	8.9 (4.2)	5.10	544	.001**	0.50	0.28, 0.65
	Subscale 3	9.2 (4)	7.8 (3.8)	4.03	544	.001**	0.37	0.19, 0.55
	TIBS scale	38.6 (13.1)	31.3(13)	6.16	544	.001**	0.56	0.38, 0.74
		Nationality, M (SD)						
		British *n* = 418	Other *n* = 134					
	Subscale 1	18.4 (6.7)	14.8 (6.2)	5.78	550	.001**	0.55	0.35, 0.75
	Subscale 2	10.6 (4.6)	9.8 (4.1)	1.71	550	.088	0.17	− 0.03, 0.36
	Subscale 3	9.0 (4.1)	8.3 (4)	1.96	550	.051	0.19	− .004, 0.39
	TIBS scale	38 (14)	32.9(13)	3.94	550	.001**	0.38	0.18, 0.57
		Sexual orientation, M (SD)						
		LGB + a, *n* = 206	Heterosexual, *n* = 359					
	Subscale 1	21.1 (6.6)	15.4 (6)	− 10.23	563	.001**	0.92	0.74, 1.1
	Subscale 2	12.4 (4.9)	9.2 (3.7)	− 8.9	563	.001**	0.78	0.6, 0.96
	Subscale 3	105 (4.3)	7.9 (3.6)	− 7.76	563	.001**	0.68	0.5, 0.85
	TIBS scale	44.01 (14.3)	32.5 (11.8)	− 1042	563	.001**	0.91	0.73, 1.1
		Marital status, M (SD)						
		Other, *n* = 205	Single, *n* = 360					
	Subscale 1	18.1(7.1)	17.1(6.6)	− 1.68	563	0.09	0.15	− .02, 0.32
	Subscale 2	11 (4.6)	10 (4.4)	− 2.44	563	.015*	0.22	.05, 0.39
	Subscale 3	9.1 (4)	8.7 (4.1)	− 1.19	563	.23	0.1	− .07, − 0.28
	TIBS scale	38.2 (14.3)	35.8 (13.6)	− 1.96	563	.051*	− 0.17	.002, .35
		Religion, M (SD)						
		Non- religious, *n* = 183	Believers, *n* = 377					
	Subscale 1	18.4 (6.7)	15.4 (6.5)	− 5.16	558	.001**	0.46	0.28, 0.64
	Subscale 2	10.7 (4.6)	9.6 (4.2)	− 2.99	558	.003**	0.26	.08, 0.44
	Subscale 3	9.2 (4.1)	8.1 (3.9)	− 3.23	558	.001**	0.29	0.11, 0.46
	TIBS scale	38.4 (13.9)	33 (13.2)	− 4.45	558	.001**	0.39	0.22, 0.57
Spain		Gender, M (SD)						
		Women, *n* = 375	Man, *n* = 162					
	Subscale 1	17.34 (6)	14.7 (6.5)	4.51	535	.001**	0.42	0.24, 0.61
	Subscale 2	11 (4.7)	8.8 (4.2)	5.36	535	.001**	0.48	0.30, 0.67
	Subscale 3	10 (3.9)	8.8 (3.9)	3.12	535	.001**	0.30	0.11, 0.48
	TIBS scale	38.3 (11.5)	32.3(12.4)	5.34	535	.001**	0.50	0.32, 0.70
		Nationality, M (SD)						
		Other, *n* = 34	Spanish, *n* = 511					
	Subscale 1	15.9 (7)	16.7 (6.3)	0.67	543	0.55	− 0.12	− 0.45, 0.23
	Subscale 2	11.5 (5)	10.35 (4.7)	− 1.30	543	0.20	0.24	− 0.1, 0.6
	Subscale 3	9.6 (4)	9.7 (3.9)	0.04	543	0.97	− 0.01	− 0.35, 0.34
	TIBS scale	37.1 (13.6)	36.7 (12.2)	− 0.15	543	0.88	0.03	− 0.32, 0.38
		Sexual orientation, M (SD)						
		LGB + *a*, *n* = 153	Heterosexual, n = 392					
	Subscale 1	20.1 (5.9)	15.3 (6)	− 8.36	543	.001**	0.79	0.6, 0.99
	Subscale 2	11.3 (4.4)	10.1 (4.8)	− 2.73	543	.001**	0.25	.07, 0.44
	Subscale 3	10.9 (4.2)	9.2 (3.7)	− 4.72	543	.001**	0.45	0.26, 0.64
	TIBS scale	42.2 (11.9)	34.6 (11.8)	− 6.78	543	.001**	0.65	0.46, 0.84
		Marital status, M(SD)						
		Other, *n* = 188	Single, *n* = 357					
	Subscale 1	16.5 (6.2)	16.7 (6.4)	0.37	543	0.71	− .03	− 0.21, 0.14
	Subscale 2	11 (5)	10.1 (4.6)	− 2.04	543	.048*	0.18	.01, 0.36
	Subscale 3	9.9 (3.7)	9.5 (4)	− 1.99	543	0.23	0.11	− .07, − 0.28
	TIBS scale	37.4 (11.9)	36.4 (12.6)	− .98	543	0.33	.09	− .09, − 0.26
		Religion, M(SD)						
		Non-religious, *n* = 156	Believers, *n* = 382					
	Subscale 1	17.2 (6.2)	15.2 (6.2)	− 3.29	536	.001**	0.31	0.12, 0.50
	Subscale 2	10.2 (4.5)	10.8 (5.2)	1.16	536	.248	− 0.12	− 0.30, .07
	Subscale 3	9.9 (3.8)	9.1 (4.1)	− 1.88	536	.062	0.18	− .003, 0.37
	TIBS scale	37.2 (11.7)	35.1 (13.1)	− 1.74	536	.083	.017	− .01, 0.37

^a^Lesbian, gay, bisexual, and + other sexual orientations not specified

^*^p ≤ .05, **p ≤ .01

The TIBS and subscale scores in both samples revealed comparable outcomes, signifying that, among the analyzed variables, women and LGBTQIA+ individuals exhibited more inclusive behaviors towards transgender people. Effect size differences varied from medium to large. Notably, the British sample demonstrated heightened positive behaviors among British citizens (vs. non-British), singles (vs. those in relationships), and non-believers (vs. religious individuals), with effect size differences ranging from small to medium.

Table 7 indicates small to medium correlations between the TIBS with all the scales, except for a large correlation between the TIBS and the EANT in the English version. Small correlations emerge between scales and age. The TIBS also exhibits medium to large correlations with its subscales across both countries. More transgender-inclusive behaviors correlate with being younger, with lower sexual risk behaviors (HPSC), and with fewer sexist (DSS), homophobic (HATH) and transphobic (EANT) attitudes.

**Table 7:** Correlations between the British and the Spanish Transgender Inclusive Behavior Scale and other criterion variables

Criterion variables																		
	1-F		2-F		3-F		TIBS		HPSC		DSS		HATH		EANT		AGE	
	UK	Spain	UK	Spain	UK	Spain	UK	Spain	UK	Spain	UK	Spain	UK	Spain	UK	Spain	UK	Spain
1-F	1	1																
2-F	.71**	.40**	1	1														
3-F	.75**	.66**	.70**	.47**	1	1												
TIBS	.94**	.88**	.87**	.74**	.87**	.84**	1	1										
HPSC	− .19**	− .18**	− .21**	− .23**	− .20**	− .20**	− .22**	− .24**	1	1								
DSS	.33**	.15**	.22**	− .02	0.22	.10*	.30*	.11*	− .05	0.01	1	1						
HATH	− .39**	− .37**	− .25**	− .16**	− .25	− .19**	− .34**	− .31**	.14**	0.01	− .47**	− .26**	1	1				
EANT	− .56**	− .36**	− .44	− .13**	− .39**	− .37**	− .53**	− .35**	.22**	0.06	− .52**	− .29**	.83**	.35**	1	1		
AGE	− .19**	− .14**	− .08	0.01	− .13**	− .08	− .16**	− .09*	− .01	.13**	− .16**	0.02	.20**	0.04	.17**	.18**	1	1

^*^p ≤ .05, **p ≤ .01

## Discussion

The study successfully achieved its objective by adapting and validating a Spanish and English version of the TIBS that adequately met the psychometric properties, aligning with the criterion variables of Kattari et al. (2018). In contrast to the one-component structure of Kattari et al. (2018), our EFA and CFA revealed a more suitable 3-factor model.

The first subscale, the Transgender Inclusive Practices Advocacy Subscale, delves into activities for acquiring knowledge about the transgender community, emphasizing proactive advocacy. The second subscale, the Gender Inclusive Language Practices Subscale, focuses on linguistic actions fostering gender respect, highlights language’s pivotal role in shaping inclusivity. The third subscale, the Transgender Inclusive Policy Awareness Subscale, evaluated awareness of local resources and policies that foster gender inclusiveness in employment, and housing, highlighting the systemic contributions to inclusivity. These subscales assist researchers, practitioners, and policymakers in assessing awareness, language practices, and the effectiveness of trans-inclusive policies in Spanish and English.

Integrating these subscales within the TIBS offers a comprehensive understanding of transgender-inclusive behaviors and a detailed analysis of intervention impacts across cultural contexts. For example, British participants showed more advocacy (subscale 1) compared to Spanish participants, who had greater policy awareness (subscale 3). Despite the lack of inclusive language norms in Spanish, such as the use of the masculine as neutral (see supplementary material for more information), no significant differences were observed in subscale 2 between the UK and Spanish participants, indicating similarities in language practices. Hence, the TIBS provides a nuanced framework for understanding transgender-inclusive behaviors across cultures.

Our study (*M* = 36.7, *SD* = 13.1) reveals less inclusive behaviors towards transgender people compared to the Kattari et al. (2018) research (*M* = 47.6, *SD* = 12.7). This difference may be related to the lower presence of gender diverse participants in the current study. Conversely, our research demonstrates higher trans inclusive behaviors than Fernández et al. (2020), whose participants had a mean age of 48.48 years (*M* = 33.2, *SD* = 12). These variations within the Spanish context could be linked to generational differences (Calvo, 2021; Cunha et al., 2021).

The TIBS demonstrates robust construct validity, with findings confirming associations between TIBS-measured inclusive behaviors and higher protective communicative behaviors (HPSC) and homophilic attitudes (HATH), along with lower levels of sexist (DSS) and transphobic attitudes (EANT). These relationships align with the importance of gender considerations in sexual education, as advocated by Calvo (2021) and Mukoro (2021). Convergent validity is established through consistent associations with HATH and EANT (Alonso-Martínez et al., 2021, 2023, 2024; Harbaugh & Lindsey, 2015; Heras & Ortega, 2020; Kaufman et al., 2019), highlighting TIBS’s unique contribution to understanding inclusive behaviors in relation to transgender issues, intimate partner communication, and sexist attitude.

The current study establishes the construct validity of gender diversity scales, revealing more inclusive behaviors in women, younger students, LGBTQIA+ individuals, and non-believers across both English and Spanish samples. In the Spanish sample, variables such as religion, nationality, and marital status align directionally with the British sample but lack statistical significance, akin to the findings of Kattari et al. (2018). These outcomes suggest potential discriminant validity for future studies. Consistent with Lee et al. (2021), Kim et al. (2024) and Paéz et al. (2015), the results link homophobic and transphobic attitudes to gender roles, sexual orientation, and religious adherence, with reduced prevalence in women, LGBTQIA+ individuals, and non-religious cohorts. These data are related to the manifestation of more hostile attitudes towards gender and roles by men compared to women (Hegarty et al., 2021; Zell et al., 2015). Variations between Spanish and British samples may stem from cross-cultural disparities.

The study reveals significant differences in configurational and metric invariance, affirming TIBS measures consistent underlying factors across countries. Although metric-scalar invariance nearly reached, scalar-metric invariance was not attained, aligning with prior research that deems achieving strict measurement invariance across diverse cultural groups is improbable (Dong & Dumas, 2020).

The similarity results on TIBS in Spain and the U.K. suggest that globalization of digital media information and adherence to European treaties contribute to advancing gender equality (Cardon et al., 2018; Spanish Youth Institute, 2019; United Nations Educational, Scientific & Cultural Organization, 2018). Attitudes toward transgender people are increasingly positive in younger populations and this is attributed to trans-positive content in social networks, education, and legislation, fostering awareness and minimizing sociodemographic biases (Cacciatore et al., 2019; Department of Education, 2022; Kim et al., 2024; Ladwig, 2023; Páez et al., 2018).

This research addresses limitations of previous studies, validating the TIBS in a different language with diverse populations. It can be used to assess the inclusive behavior acquired through educational interventions and measure the legislative impact of policy changes. This scale helps to identify the areas in which the population can improve their inclusive acts to provide more support to the transgender community.

The validated scales included samples of future professionals across sectors, aligning with the observation of Kattari et al. (2018) that transgender students in secondary and higher education encounter more discrimination than their non-student counterparts. University students, pivotal in shaping transgender-inclusive behaviors, underwent initial validation for subsequent interventions. Upon graduation, their influence extends to diverse domains such as education, finance, labor resources, and healthcare (Day et al., 2019; Haley et al., 2019; Schucan & Pitman, 2020). While over 70% of Spain and the UK’s population pursue university studies (Eurostat, 2020), caution is needed in generalizing these findings.

### Limitations

While the scale used is validated for Spanish-speaking populations, applicability constrains between Spain and Latin America exist. The ongoing debate on inclusive language, especially in Latin languages like Spanish, poses challenges. The Royal Spanish Academy favors masculine neutrality, prompting academic discourse on gender-neutral terms (Calvo, 2021; Cunha et al., 2021). The study recommends refining trans inclusive actions, emphasizing individual and contextual differences, advocating for a nuanced understanding, and endorsing multifaceted approaches to foster true acceptance within the transgender community.

Future research should consider these limitations, conducting replications in diverse populations and settings, including participants from the general public opinion. Translation into various languages should be pursued to enhance instrument standardization. Evaluating scores post-modifications to sex education in the U.K. and Spain following legislative changes is essential, with a recommendation to include variables such as ideology and self-esteem.

### Conclusion

The TIBS has excellent reliability and validity and proves its innovation in diverse cultural contexts like the UK and Spain. As a tool, it enhances understanding of some transgender inclusive behaviors toward transgender people taken individually, which can be influenced by both policy frameworks and structural factor. With specialized subscales providing a nuanced analysis, researchers can tailor assessments to their context, potentially evaluating interventions and measuring social transformations’ impact effectively.

## Electronic supplementary material

Below is the link to the electronic supplementary material.

Supplementary file1 (DOCX 26 KB)

## Data Availability

The data reported in this manuscript can be approached by contacting the corresponding authors and it is deposited in a digital repository that can be accessed from the following link, https://doi.org/10.5281/zenodo.5939452

## Appendix 1

The Transgender Inclusive Behavior Scale.

**Table:**

No	Items
1	I ask for pronouns when I meet someone new
2	I use gender neutral language to refer to people whose pronoun I do not know
3	I ensure spaces where I host/attend events offer gender-neutral bathrooms
4	I use the terms “non-transgender” or “cisgender” to refer to people whose sex they were assigned at birth matches their current gender identity
5	I have participated in discussions about the effects and/or benefits of cisgender or non-transgender privilege
6	I share my pronouns when I introduce myself to someone new
7	I have asked my friends, co-workers, and/or family members what their pronouns are
8	I read books/blogs/articles by transgender women, transgender men, and gender non-conforming individuals
9	I speak out against “womyn born womyn” or transgender exclusive policies (such as those used by Michigan Women’s Festival)
10	I initiate conversations about how my community can support transgender individuals
11	I try to keep myself updated on ongoing conversations about acceptable language to use when referring to transgender individuals
12	I work to educate myself on issues regarding transgender communities
13	I am aware of local resources that offer support to transgender people
14	I keep myself updated on whether employment policies in my state/city include transgender people
15	I keep myself updated on whether housing policies in my state/city include transgender people

Likert scale of 5 answers: 1 (Never), 2 (Rarely), 3 (Sometimes), 4 (Often) and 5 (Always).
