# Supplementary material for: Validation in Spanish and English of the Transgender Inclusive Behavior Scale
Source: Arch Sex Behav. 2024 Sep 19;53(10):3769–84. doi: 10.1007/s10508-024-02982-7 (PMC11588774; doi:10.1007/s10508-024-02982-7)
Supplement: Supplementary file 1 — Supplementary file1 (DOCX 26 KB) [file 10508_2024_2982_MOESM1_ESM.docx]

**SUPPLEMENTARY MATERIAL**

**Table S1.**

*The Inclusive Behaviors towards Trans People Scale in Spanish (Escala de Comportamientos Trans Incluyentes, TIBS).*

| **Nº** | **Items** |
| --- | --- |
| 1 | Cuando conozco a una persona le pregunto su nombre y/o pronombre personal antes de utilizar estos pronombres asociados a un género determinado. |
| 2 | Procuro usar lenguaje inclusivo cuando me refiero a una persona cuyo nombre y pronombre personal no conozco. |
| 3 | Me aseguro de que los espacios donde organizo/asisto a los eventos ofrezcan baños inclusivos de género. |
| 4 | Cuando tengo una conversación en la que se mencionan las identidades de género uso el término "cisgénero" para referirme a las personas cuyo sexo asignado al nacer coincide con su género. |
| 5 | He participado en discusiones sobre las consecuencias y/o beneficios de los privilegios de las personas cisgénero. |
| 6 | Comparto mi pronombre personal y/o el nombre por el que quiero que me identifiquen cuando me presento a una persona que no conozco. |
| 7 | Le he preguntado a mis amigos, compañeros de trabajo y/o miembros de mi familia con que pronombre personal y nombre prefieren que les llame. |
| 8 | Leo libros/blogs/artículos o contenido multimedia relacionados con la identidad de género de personas que manifiesten ser transgénero o individuos con género no binario. |
| 9 | Hablo en contra de las políticas de género que excluyen a las personas transgénero (como las que apoyan el feminismo radical trans-excluyente). |
| 10 | Inicio conversaciones sobre cómo mi comunidad (mi círculo de amistades, etc.) puede apoyar a las personas transgénero y no binaries. |
| 11 | Intento mantenerme actualizado/a sobre las conversaciones que se están desarrollando acerca del uso de un lenguaje inclusivo. |
| 12 | Estoy intentando educarme en temas de identidad de género relacionados con las personas trans. |
| 13 | Estoy concienciado/a sobre las ayudas y recursos locales que se ofrecen en apoyo a las personas trans. |
| 14 | Me mantengo informado/a sobre si las políticas estatales de empleo incluyen a las personas transgénero y no binaries. |
| 15 | Me mantengo informado/a sobre si las políticas estatales de vivienda incluyen a las personas transgénero y no binaries. |

Escala Likert de 5 respuestas: 1 (Nunca); 2 (Raramente), 3 (A veces); 4 (A menudo) y 5 (Siempre).

**Scale explanations**

This scale has been validated for use among Spanish-speaking populations, but it is important to note that there may be limitations in its applicability due to the different contexts between Spain and Latin America. However, the continuous debated about this topic and the changes in vocabulary related to inclusive language are also considered a constrain, which adds to the difficulty posed by Latin languages, such as Spanish, which has gender-determined words. The most appropriate way to achieve linguistic inclusion in countries with Latin languages is currently under debate (Calvo, 2021; Cunha et al., 2021). The Royal Spanish Academy currently upholds the masculine form as neutral, aiming for linguistic coherence across Spanish-speaking nations. Some academics oppose inclusive language, while others advocate for incorporating both feminine and masculine endings, gaining traction in academia for gender-neutral terms. Critics argue the proposals are incomplete, lacking inclusivity for all gender expressions and suggest other proposals such as adding the "e" ending to gendered words (Calvo, 2021).

The selection of actions considered trans inclusive, such as inquiring about pronouns, could be narrowed down to asking whether individuals respect others' chosen names in various scenarios. This suggests that those utilizing this framework should account for individual differences and social contexts. It underscores the need for a nuanced understanding, acknowledging that a reluctance to embrace specific behaviors does not necessarily indicate a lack of support and might be linked to hostile environments where individuals may feel unsafe talking about these matters. The study advocates for a comprehensive approach, recognizing diverse perspectives within the transgender community and cautioning against imposing universal expectations. It argues for the need to employ multifaceted approaches to truly foster acceptance. Considering this framework, the TIBS and their subscales are a tool to assess the frequency and the knowledges of some of these trans inclusive behaviors in society, encouraging to evaluate this construct with more perspectives.
